# Forensic neuropathology: 2026 update

**DOI:** 10.17879/freeneuropathology-2026-9343

**Published:** 2026-05-18

**Authors:** Jakob Matschke

**Affiliations:** 1 Institute of Neuropathology and Forensic Neuropathology Unit, Institute of Neuropathology & Institute of Forensic Medicine, University Medical Center Hamburg-Eppendorf, Hamburg, Germany

**Keywords:** Forensic neuropathology, Neurotraumatology, Abusive head trauma, Traumatic brain injury, TBI, Intimate partner violence, IPV, Chronic traumatic encephalopathy, CTE, Sudden unexpected death in epilepsy, SUDEP, Sudden infant death syndrome, SIDS

## Abstract

This review discusses key publications in forensic neuropathology from the 2025 literature. In abusive head trauma (AHT), biomechanical models help clarify injury mechanisms, particularly the role of sagittal angular acceleration in younger infants. However, challenges remain, such as detecting subtle brainstem pathology with neuroimaging, stressing the need for prospective clinicopathological correlation. Meanwhile, understanding of chronic traumatic encephalopathy (CTE) is expanding beyond contact sports to include victims of intimate partner violence. Research on sudden unexpected death in epilepsy (SUDEP) indicates overlaps with sudden infant death syndrome (SIDS) and identifies key risk factors. At the same time, forensic neuropathology's primary role remains the exclusion of other causes of death. Artificial intelligence shows promise for analyzing radiological and histopathological data in traumatic brain injury (TBI) and epilepsy, though its adoption for routine purposes remains a distant goal. Further developments include a better understanding of the cerebellum's vulnerability to TBI, standardized postmortem MRI protocols, and the use of cerebrospinal fluid biomarkers, such as GFAP and S100B, to estimate time of death. Finally, research continues to probe the complex links between brain morphology and behavior, and recent studies of the neuropathology of alcohol use disorder have revealed microglial changes rather than overt neuronal loss.

## Introduction

**Figure 1:**
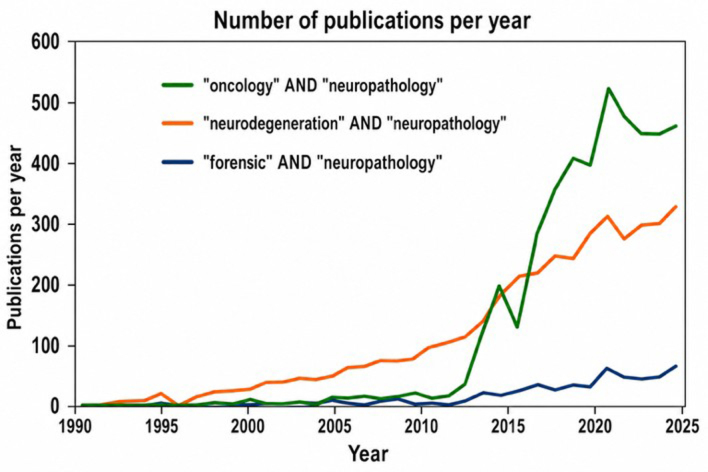
Number of publications per year for ''oncology'' AND ''neuropathology'' (green line), ''neurodegeneration'' AND ''neuropathology'' (orange line), and ''forensic'' AND ''neuropa-thology'' (blue line), based on a Pub-Med search by the author.

At first glance, neuropathology and forensic pathology seem like strange bedfellows. Neuropathology involves detailed analysis of minute tissue specimens using advanced techniques such as histopathology, immunohistochemistry, methylation profiling, and next-generation sequencing. Founded by eminent scientists such as Jeann-Martin Charcot, Alois Alzheimer, and Santiago Ramón y Cajal around the turn of the last century, neuropathology has evolved from a niche specialty into a prominent scientific discipline, contributing significantly to research in neuro-oncology, neurodegeneration, and beyond. Forensic pathology, by contrast, focuses on identifying the cause and manner of death through macroscopic findings and case-based judgment. This reliance sometimes led to concerns about objectivity, reproducibility, and scientific rigor [1]. Despite these differences, forensic neuropathology has emerged as an interdisciplinary field, combining the strengths of both disciplines to produce insights neither could achieve on its own. The publication of several authoritative textbooks and the establishment of continuing medical education programs, such as those by the European Confederation of Neuropathological Societies (EURO-CNS), reflect this development. However, compared with the major workhorses in neuropathology (neuro-oncology, neurodegeneration, etc.), forensic neuropathology still produces significantly fewer publications (**Figure 1**). Therefore, this 2025 update is intended as a practical resource, presenting key literature to support neuropathologists in forming expert opinions that require contextual interpretation. This process requires neuropathologists to engage comprehensively with each case's clinical background, ancillary results, and forensic findings to build a coherent narrative. The publications presented are based on the author's personal selection through regular, standardized searches of medical and scientific databases (Web of Science, PubMed) using individual keywords or combinations thereof, such as "neuropathology", "neurotraumatology", "abusive head trauma", "forensic pathology", "traumatic brain injury", etc.

## 1. Abusive Head Trauma in infants: biomechanics

Neuropathologists play a key role in evaluating alleged cases of abusive head trauma (AHT) in infants, a syndrome of injuries officially defined as resulting from blunt impact or violent shaking, though often equated informally with shaken baby syndrome [2]. Main neuropathological findings in AHT include hypoxic-ischemic encephalopathy (HIE), traumatic axonal injury (TAI), and subdural and retinal bleedings (SDB, RB). However, AHT faces significant challenges because of ongoing scientific controversy, diagnostic uncertainty, and substantial legal implications. Since its medical discovery in the 1970s, the concept of AHT has met with persistent criticism and skepticism, with some even denying its very existence [3,4]. Subsequently, neuropathologists serving as expert witnesses must not only be trained to identify syndromic injuries, their morphology, and diagnostic criteria, but also have a comprehensive and critical understanding of the scientific discourse, given the considerable legal and ethical consequences of their testimony, especially when facing skilled defense attorneys.

A major issue inherent to the concept of AHT is the obvious lack of randomized controlled trials. In addition, up to the date of this writing, no independent witness has ever observed a previously healthy infant being shaken and then displaying AHT-typical symptoms or findings. To address these limitations, biomechanical studies have been part of AHT research since early on [5]. However, the mathematical and physical modeling of AHT is extraordinarily challenging for non-professionals, as it requires integrating nonlinear dynamics, sophisticated finite-element analysis of the brain's viscoelastic properties, and more to simulate the rotational and translational forces during a shaking event.

Hutchinson et al. systematically reviewed studies published from 2017 to 2023 that model AHT to help medical professionals understand these models [9]. Using PRISMA guidelines, they identified 2 animal, 5 physical, and 10 mathematical modeling studies. Both animal studies used rodents; their relevance to human shaking trauma seems uncertain. Physical models explored head kinematics during shaking, while mathematical models focused mainly on the eye. Notably, most injury thresholds and tissue properties were scaled from adult or animal data, and shaking inputs were largely oversimplified. Therefore, the authors argue that future work should focus more on realistic shaking inputs and develop validated injury thresholds specific to infants (the latter will certainly be extremely difficult to implement).

**Figure 2:**
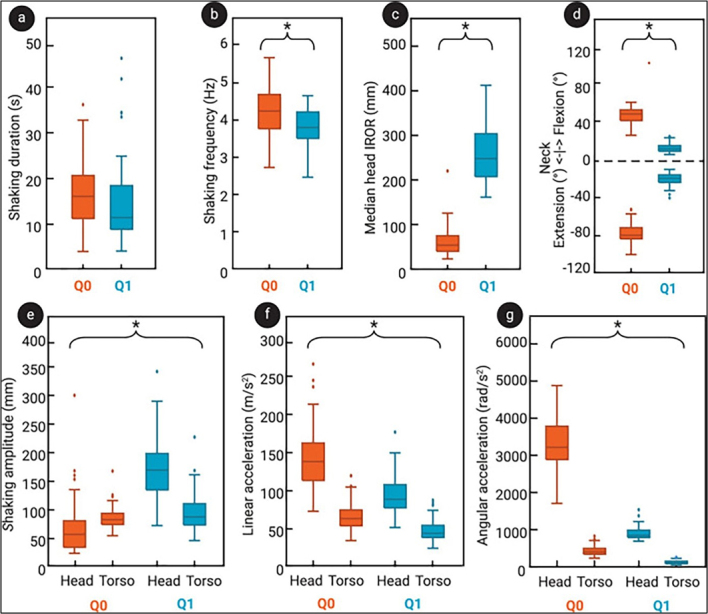
Results from experiments on shaking a 6-week-old (Q0) and a 1-year-old infant (Q1) surrogate from the study by Hutchinson et al. [10]: Distributions of shaking duration (**a**) and shaking frequency (**b**) across all trials; the median head IROR (**c**) for the fiercest cycles across all trials, and the peak neck flexion and extension across all trials (**d**). Shaking amplitude (**e**), linear acceleration (**f**), and angular acceleration (**g**) are shown for both the head and the torso. [Note by this author: The IROR is, in technical terms, the distance between the ICOR (i.e., the point in space around which an object's motion can instantaneously be described as a pure rotation) and the center of mass of the object. In plain English, that roughly translates to: The IROR is the distance between the ICOR (the exact spot the infant's head is spinning around at any given moment) and the center of the head]. Reproduced under the Creative Commons Attribution 4.0 International License (http://creativecommons.org/licenses/by/4.0/).

Consequently, Hutchinson et al. published an interesting study comparing the kinematics of shaking a smaller dummy (as a surrogate for a 6-week-old) and a larger dummy (as a surrogate for a 1-year-old) [10]. Both dummies contained a single-axis gyroscope in the head and a single-axis gyroscope in the torso, as well as one tri-axial accelerometer in the back of the torso. The dummies were placed in a standardized posture in a frame, and participants shook them as violently as possible twice, once while sitting and once while standing. A motion capture system with twelve infrared cameras recorded the positions of reflective markers mounted to the head and torso of each dummy. Participants generated higher head and torso accelerations when shaking the smaller dummy than when shaking the larger one. Additionally, higher peak sagittal angular accelerations were associated with smaller rotational radii in the younger dummy; these mechanisms are considered a main factor in producing typical AHT findings (**Figure 2**). The authors conclude that, since sagittal angular acceleration is regarded as a key mechanism in AHT, their findings show that shaking a younger or smaller infant produces kinematics more consistent with those believed to cause injury. These results are consistent with the epidemiology of AHT, which mainly occurs in infants aged 2–4 months.

## 2. Abusive Head Trauma in infants: encephalopathy

Aside from the challenges of interpreting and evaluating findings considered typical of AHT, an important question remains unresolved: the actual mechanisms of death. Analysis of statements by confessing perpetrators, which obviously should be regarded as scientifically valid only with the strongest reservations, suggests that an infant subjected to severe shaking will suffer respiratory and/or cardiac arrest within seconds [11]. Most experts consider this the cause of HIE, which, via accompanying massive brain swelling, ultimately proves fatal. Yet the exact pathophysiological mechanism of this collapse remains incompletely understood. The prevailing hypothesis, also endorsed by this author, posits a focal traumatic axonal injury, in a proportion of cases demonstrable on immunohistochemical examination, involving the lower brainstem in the vicinity of autonomic cardiovascular and respiratory regulatory nuclei. Despite its anatomical and pathophysiological plausibility, this hypothesis has not yet been conclusively demonstrated [14].

**Figure 3:**
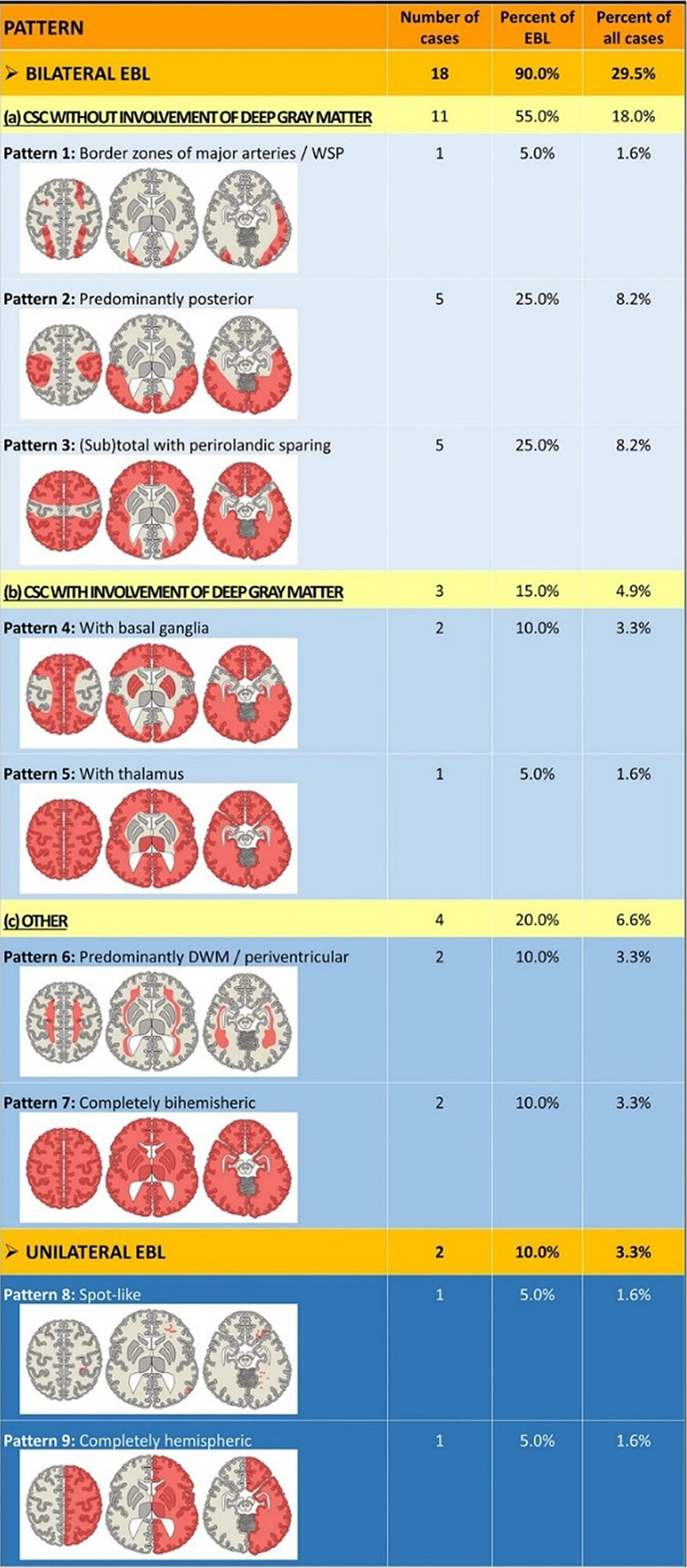
Schematic illustration of a classification system for extensive brain lesions (EBL) in AHT proposed by Hahnemann et al. [15]. A total of 9 EBL patterns can be subdivided into 7 bilateral and 2 unilateral EBL patterns. The bilateral EBL can be further subdivided into 3 patterns without involvement of the deep gray matter, 2 with involvement of the deep gray matter, and 2 additional "other" patterns. The unilateral EBL pattern includes a spot-like and a hemispheric pattern, the mechanisms of which remain elusive (abbreviations: CSC, cortico-subcortical; DWM, deep white matter; WSP, watershed pattern). Reproduced under the Creative Commons Attribution 4.0 International License (http://creativecommons.org/licenses/by/4.0/).

**Figure 4:**
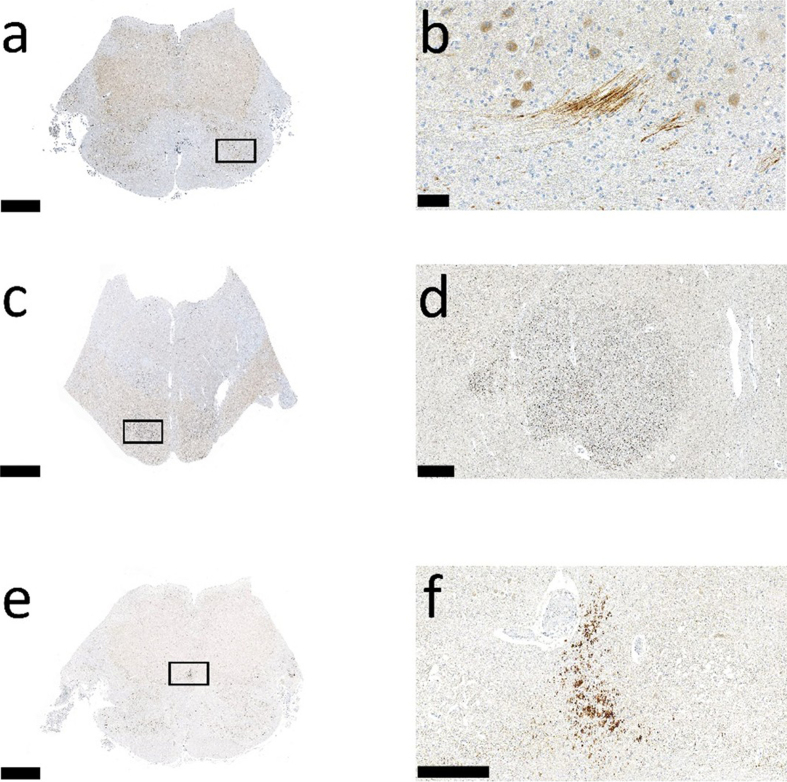
Different patterns of APP-positive localized traumatic axonal injury in the lower brainstem in three infantile AHT-victims. Scale bar in **a**, **c**, and **e**: 2.5 mm; scale bar in b: 50 μm, in **d**: 500 μm, and in **f**: 250 μm; all cases from the author's personal archive)

Hahnemann et al. reported the neuroradiological prevalence and features of brain lesions associated with AHT, aiming to establish a classification system [15]. In their retrospective multi-center study, 61 AHT victims were examined (**Figure 3**). The authors distinguished focal brain lesions (FBL) from extensive brain lesions (EBL) that extend across more than one lobe of the brain. Quite surprisingly, lesions of any type were found in only 31 of 61 cases (50.8 %)—possibly because the study population mainly included infants who survived. The incidence of FBL reported in this study—relatively high compared with the existing literature, at 15 cases (24.6 %)—can be attributed to the authors' broad definition, which included not only contusions as the classical FBL associated with blunt head injury (identified in only 1 of 61 cases), but also rather non-specific findings such as "congestive hemorrhages", or lacerations, which are thought to result from diffuse shear injury (similar to the so-called gliding contusions [16]). The study found no localized brainstem lesions, questioning the link between HIE and localized axonal damage in the brainstem. Notably, most patients survived, and neuropathological evidence suggests that focal brainstem lesions may be too small for detection by neuroradiology (**Figure 4**). This discussion makes it clear that prospective studies detailing the correlation between neuropathology and neuroradiology are urgently needed.

## 3. Chronic traumatic encephalopathy (CTE) and intimate partner violence (IPV)

The effects of repetitive traumatic brain injury (TBI) have been recognized for nearly a century, historically described as "punch drunk syndrome" or "dementia pugilistica", now understood as chronic traumatic encephalopathy (CTE) [17]. Interest in CTE increased substantially after forensic neuropathologist Bennet Omalu published findings from the brain of former National Football League player Mike Webster, who died in 2002 with severe neurodegenerative decline [20]. Since then, numerous studies have advanced the field, including the development of standardized micromorphological diagnostic criteria for CTE and a report on CTE-equivalent findings in a naturally occurring animal model [21].

**Figure 5:**
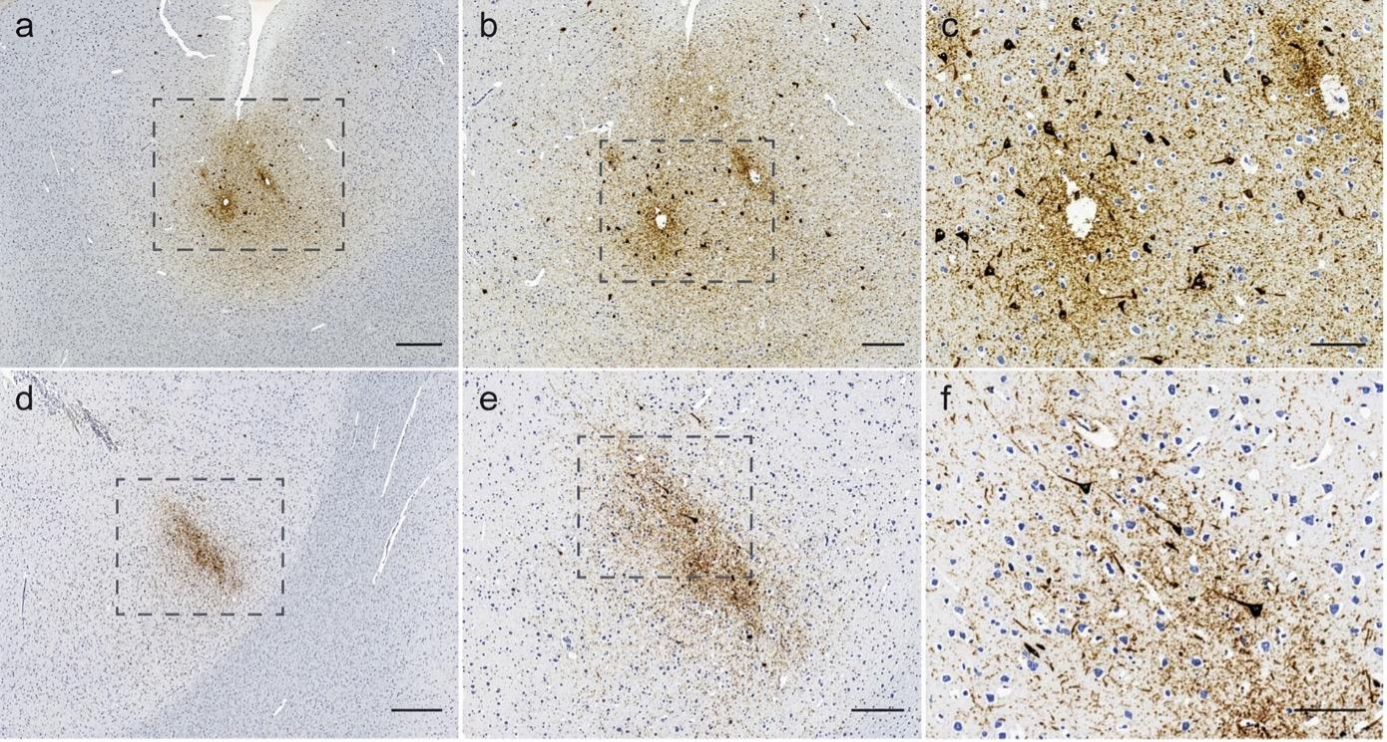
Chronic traumatic encephalopathy (CTE) following intimate partner violence (IPV), taken from [26]. Case #1 (panels **a–c**) shows representative tau immunostaining in a female who died in her 5^th^ decade after more than 20 years of IPV, with more than 15 recorded head injuries and more than 30 assault-related medical presentations. Case #2 (panels **d–f**) is from a female who died in her 5^th^ decade after 17 years of IPV, with more than 20 documented head injuries and more than 40 assault-related medical presentations. Reproduced under the Creative Commons Attribution 4.0 International License (http://creativecommons.org/licenses/by/4.0/).

In 1990, Roberts et al. reported a case of "dementia in a punch drunk wife" who died at age 75 years after years of repeated violence by her husband; since the report included only low numbers of paired helical filament immunoreactive tangles in the frontal cortex, it remains unclear whether this might indeed have been CTE due to intimate partner violence (IPV) [24]. The first definitive case report on CTE in a young victim of repeated IPV was published in 2021 [25], followed by further cases [26], sparking hope for a robust biomarker that could reliably indicate repeated IPV and meet the rigorous standards required for legal evidence in court (**Figure 5**). However, a recent larger exploratory study of 14 patients (all female) with a history of IPV did not identify any cases of CTE [27], although this is almost certainly attributable to the composition of the study population, which obviously did not include any cases with definitive repetitive TBI.

Tiemensma and Byard comment on these findings, emphasizing the need for further research and suggesting that discrepancies across studies might reflect cohort differences [28]. They also note that some questions about CTE pathogenesis remain unanswered; apart from the classical model of repetitive TBI with roughly equivalent contributions by linear acceleration and rotational forces [29], it may involve cumulative minor trauma, high-impact forces, or a combination. Resolving these questions, particularly whether CTE in humans can arise additively or requires a force threshold, would require careful study of the timing, nature, and dose of prior head impacts in victims of inflicted trauma, especially in the context of domestic violence. Grouping IPV cohorts by documented strike frequency, force, and duration would theoretically help to clarify thresholds for CTE development. Finally, standardizing the quantification of exposure variables, such as strike intensity and frequency, would facilitate a shift from anecdotal to empirical evidence. Alas, the precise quantification of all these variables seems nearly impossible in humans within a real-world context.

Selmanovic et al. [30] reported findings from a cohort of 47 donor brains in the Late Effects of TBI Project (LETBI; [27,31]). These donors had a range of head trauma exposure patterns, including repetitive TBI, isolated i.e. single TBI, or a combination of both. Definite CTE neuropathological findings were identified in 7/47 (14.9 %), with six having a clear history of significant, repetitive TBI (including American football, boxing, military combat training, child abuse, and multiple, various accidents). Notably, at least four individuals without CTE pathology had high levels of repetitive head impact exposure, suggesting the influence of mitigating factors and underscoring the need to identify them. Consistent with previous literature, isolated TBI was rarely associated with CTE pathology in this cohort.

## 4. Sudden unexpected death in epilepsy (SUDEP)

Epilepsy, a chronic neurological disorder affecting over 65 million people worldwide, carries a significant yet widely underappreciated mortality risk. Patients face an elevated all-cause mortality risk and may die from indirect causes, such as accidents and comorbid conditions, or from direct causes, such as status epilepticus or SUDEP (Sudden Unexpected Death in Epilepsy). SUDEP is defined as the sudden, non-accidental, witnessed or unwitnessed death of a person with epilepsy, with no other cause of death found upon post-mortem examination [32]. The primary and most definitive role of neuropathology in SUDEP is to rule out other causes of sudden death; SUDEP is, by definition, a diagnosis of exclusion. Yet the neuropathologist might identify a previously unknown underlying epileptogenic condition. In addition, neuropathology helps lay the foundation for SUDEP research through standardized examination. The neuropathologist seeking to understand this mysterious condition might find three papers from 2025 helpful.

Sharma et al. published a review on the striking similarities among SUDEP, sudden unexpected infant death (SUID), with its most common phenotype, the "classical" sudden infant death syndrome (SIDS), and sudden unexplained death in childhood (SUDC) [33]. The authors discuss recent research indicating significant clinical, neuropathological, and genetic similarities among SUID, SUDC, and SUDEP. Shared characteristics include sleep-related death and prone positioning; recently, pathological variants in genes encoding voltage-gated sodium channels linked to epilepsy or cardiac arrhythmia have been shown to be enriched in SUDEP, SUDC, and SUID [34,35]. For the neuropathologist, discussing the nature and impact of subtle abnormalities in the hippocampus and/or dentate gyrus is particularly important [36]; these abnormalities, along with other hippocampal maldevelopments, have been documented in SUDC and SUDEP [39]. While it remains unclear whether these abnormalities cause seizures or result from them, their presence in children without any clinical seizure history is a significant observation.

Ochoa-Urrea and colleagues reported results from a prospective, multi-center, observational cohort study of more than 2500 children and adults with epilepsy undergoing prolonged video-EEG monitoring [40]. Baseline demographic, clinical, and cardiorespiratory data were collected, and long‑term follow‑up was conducted through clinic visits, electronic health record reviews, and telephone interviews to track seizure frequency, medication status, and mortality. The primary endpoint was time to SUDE. Among the participants, 38 (1.54 %) died from SUDEP, resulting in an SUDEP mortality rate of 4.76 cases per 1,000 person‑years. Significant predictors of increased SUDEP risk included living alone, three or more generalized convulsive seizures in the prior year, and longer ictal and postictal central apnea.

**Figure 6:**
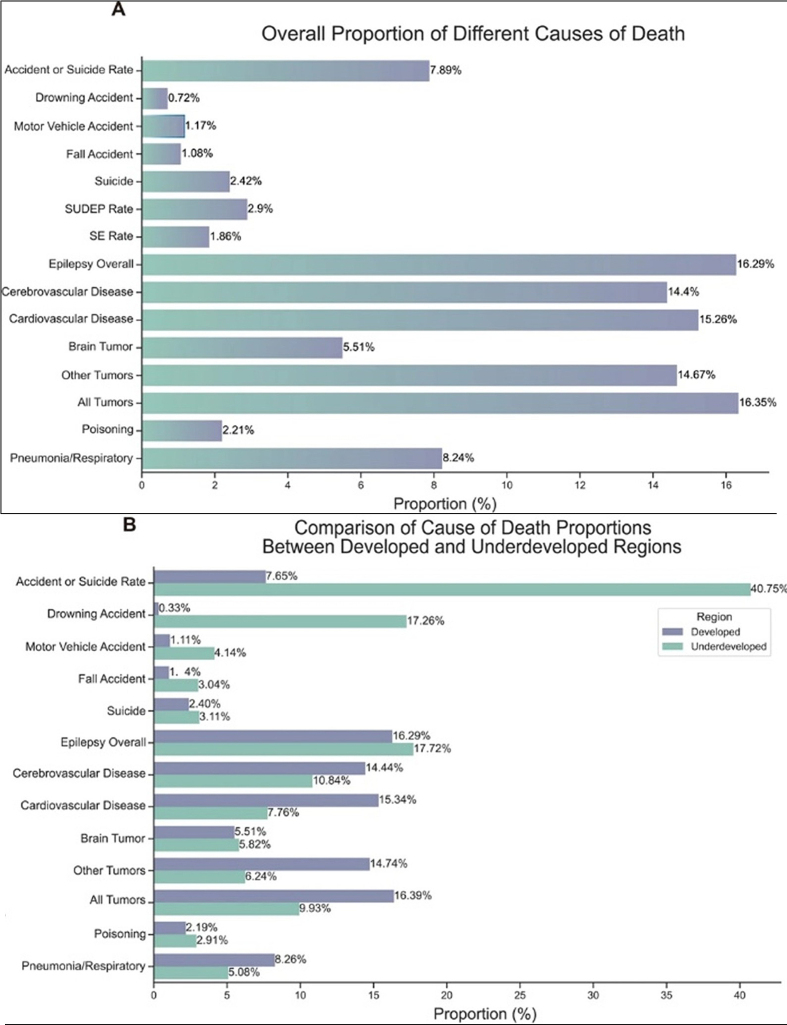
Comparison of causes of death from the study of Xu et al. on mortality in patients with epilepsy [41]: Comparison of data from all studies (in **a**) and comparison of causes of death between high-income and low-income regions (in **b**). Reproduced under the Creative Commons Attribution 4.0 International License (http://creativecommons.org/licenses/by/4.0/)

Xu et al. published a systematic review on epilepsy-related mortality [41]. Their findings revealed a gradual global decline in epilepsy mortality and highlighted critical demographic and clinical determinants of epilepsy mortality (**Figure 6**). Notably, deaths directly related to epilepsy (including SUDEP and status epilepticus) accounted for 16.29 % of all mortality. The leading causes of death among patients with epilepsy were tumors (16.35 %) and cardiovascular diseases (15.26 %), followed by accidents and suicides (7.89 %). Geographically, high-income regions showed higher mortality from tumors (16.39 % vs. 9.93 %) and cardiovascular disease (15.34 % vs. 7.76 %). In comparison, low-income regions had a significantly greater proportion of deaths from accidents and suicides (40.75 % vs. 7.65 %, with drowning alone at 17.26 %). Epilepsy-related mortality was slightly higher in low-income regions (17.72 % vs. 16.29 %).

Finally, a highly interesting philosophical essay on SUDEP and the challenges of communicating about risks and care with a delicate balance among awareness, psychological sustainability, and a genuine therapeutic alliance, even while acknowledging that knowing does not always mean being able to prevent, has been published by Di Gennaro [42].

## 5. Artificial Intelligence in forensic neuropathology

The steady rise of artificial intelligence (AI) in medicine over the last few years has led to notable advances in both diagnostics and patient care [43]. In pathology, AI is increasingly used to analyze histopathological images, detect subtle cellular changes, and even predict disease outcomes [46,47]. In neuropathology, AI has shown promising potential in neuro-oncology and neurodegeneration [48]. Consequently, numerous studies discuss the application of AI in forensic pathology and forensic neuropathology, although these fields remain relatively new and are still developing.

In 2025, a review of the current state of AI in forensic neuropathology yielded somewhat discouraging results: Treglia et al. [52] conducted a systematic review of the literature on AI in forensic neuropathology, following the Preferred Reporting Items for Systematic Reviews (PRISMA) guidelines. In total, 34 studies were retrieved from January 2014 to May 2024 and grouped into two categories: those focused on traumatic brain injury (TBI) and those centered on epilepsy. For TBI, only six studies (all based on radiological data) were identified that investigated the use of machine learning and convolutional neural networks (CNNs) on post-mortem computed tomography (PMCT) or magnetic resonance imaging (MRI). It is not overtly surprising that these studies found that CNNs can accurately distinguish fatal head injuries from uninjured cases on PMCT scans. In MRI-based detection of cerebral microbleeds in patients with TBI, the reviewed studies showed that AI models can quickly and accurately identify them. One study found that a standard deep learning CNN model was more accurate at diagnosing fatal ICB than a more advanced model (DenseNet). The studies on epilepsy and related issues, including tumor subtype differentiation, comprised a total of 28 studies. Drawing extensively on findings from studies of digitized histopathology specimens, the authors concluded that AI-assisted histopathology demonstrates high accuracy in detecting and classifying tumors and their subtypes. In addition, deep learning techniques could classify malformations of cortical development and distinguish focal cortical dysplasia from tuberous sclerosis with over 90 % accuracy. These results suggest that AI might provide objective, reproducible evidence in forensic neuropathology, supplement classical postmortem brain investigations, and enhance disease classification. At present, however, it seems, to put it cautiously, rather unlikely that such elaborate and technically challenging applications will enter the routine diagnostic workup in forensic neuropathology.

## 6. The cerebellum in forensic neuropathology

For the forensic neuropathologist, the cerebellum is not just a "little brain" but also a "witness box". The cerebellum may indicate toxicity from anti-epileptic drugs (valproic acid) or alcohol (atrophy of the upper vermis due to associated thiamine deficiency), and cerebellar herniation is an immediately obvious cause of death in severe brain swelling. Apart from severe diffuse acceleration-deceleration injury, in which the cerebellar peduncles can show traumatic axonal injury, the cerebellum's role in traumatic brain injury (TBI) has historically been viewed as that of a mere bystander [53]. The last few decades have seen a shift in this approach, recognizing that the cerebellum may be vulnerable to supratentorial TBI even when it is not directly affected. Notably, supratentorial TBI may cause cerebellar atrophy many years after the initial impact, especially in children [54].

To further appreciate the cerebellum's role in forensic neuropathology, Sivalingam et al. in 2025 review the cerebellum "for forensic and clinical neuroscience" using a broad approach [58]. In addition to discussing pathomorphological findings that forensic neuropathologists should be aware of, the review includes further sections on emerging therapies for cerebellar disorders, advances in genetic diagnosis and treatment, and many other topics that do not have an immediate impact on forensic neuropathology. The authors further summarize recent advances that increasingly recognize the cerebellum's importance and expand its role beyond motor function. Advanced neuro-imaging and molecular techniques now enable precise detection of cerebellar damage from trauma, neurodegeneration, or intoxication, offering new insights into the cause and circumstances of death.

## 7. Postmortem radiology for forensic neuropathology

**Figure 7:**
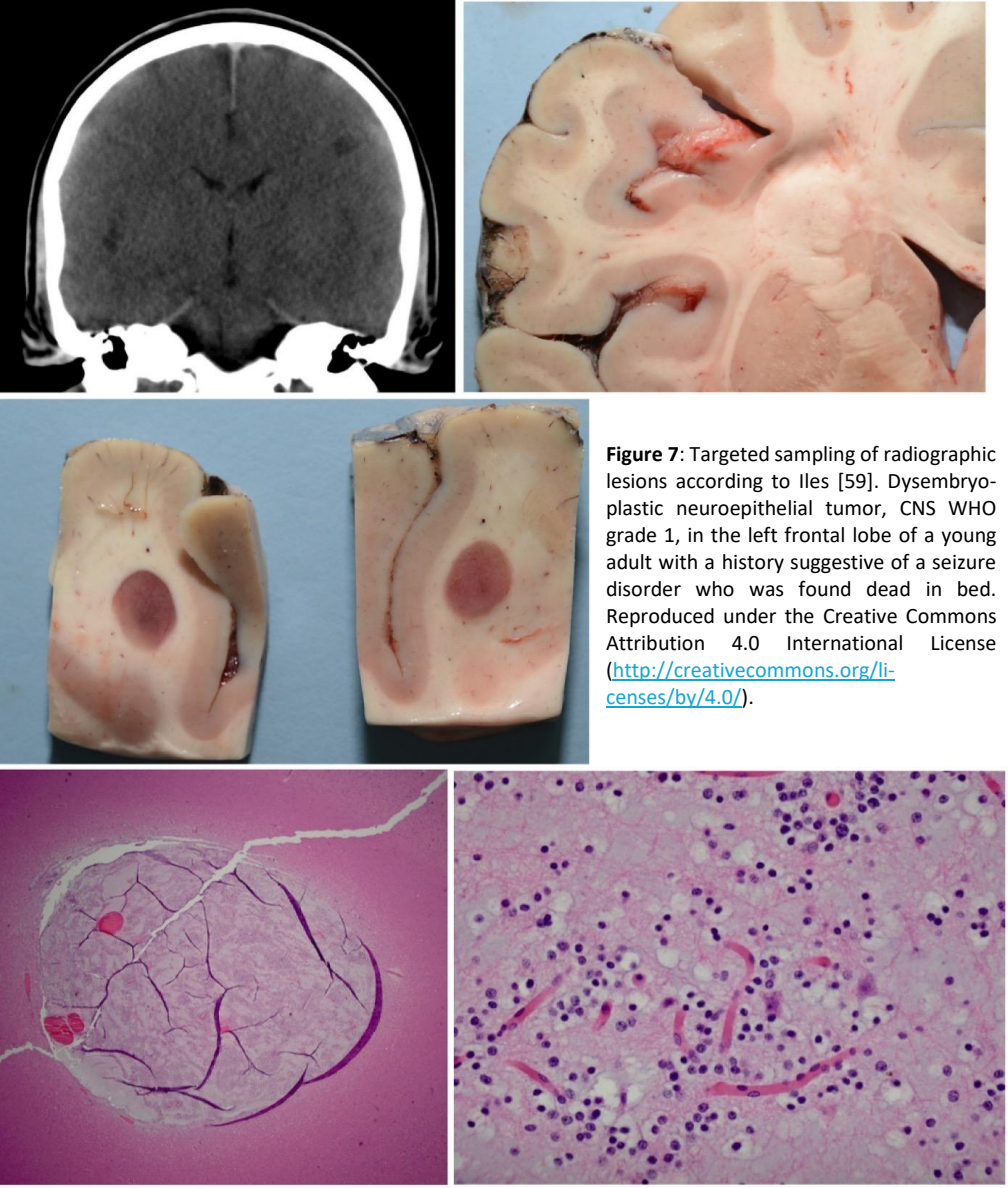
Targeted sampling of radiographic lesions according to Iles [59]. Dysembryoplastic neuroepithelial tumor, CNS WHO grade 1, in the left frontal lobe of a young adult with a history suggestive of a seizure disorder who was found dead in bed. Reproduced under the Creative Commons Attribution 4.0 International License (http://creativecommons.org/licenses/by/4.0/).

Iles reviews the role of postmortem radiological imaging in forensic neuropathology, focusing primarily on postmortem computed tomography (PMCT), and devoting only a single short paragraph on postmortem magnetic resonance imaging (PMMR) [59]. The article describes the strengths and weaknesses of these technologies in investigating sudden deaths, traumatic brain and spinal cord injuries, and other medicolegal cases. Reviewing post-mortem imaging before examination helps plan the neuropathological approach and allows for targeted sampling of radiographic lesions (**Figure 7**). PMCT is highlighted as one of the most important developments in forensic pathology over the last 25 years, as it provides a non-invasive method for documenting and analyzing injuries and pathological findings. The article emphasizes the combination of radiological imaging and traditional neuropathological examination as the future gold standard in forensic neuropathology. Since the first PMMR was reported in 1990 [60], PMMR has been increasingly used to assess unexpected deaths and fetal malformations. Although numerous protocols have been published over the past two decades, early versions were often too long (up to 90 minutes) for routine clinical use due to scanner availability and time constraints. Therefore, the European Society of Pediatric Radiology (ESPR) addressed this by endorsing a standardized 30–60-minute protocol via expert consensus in 2021 [61].

In 2025, members of the ESPR updated the 2021 discussion [62]. An international expert panel of 19 specialists from 17 centers agreed on two standard PMMRI protocols: The "minimal" protocol (under 30 minutes) uses 3-D scans of the brain and body for a quick overview. The "ideal" protocol (when there is more time) includes scans of the entire body including limbs and more detailed brain scans to detect bleeding or other issues. The "minimal" protocol includes 3-D T2 and T1 brain and body scans. The "ideal" protocol includes whole-body 3-D scans and specialized brain scans for bleeding (SWI) and tissue details (DWI). Although the expert group, according to the authors, was small and lacked members from some continents, their advice still offers useful guidance for this specialized field.

## 8. Estimating the time of death using cerebrospinal fluid

Estimating the time of death, or the post-mortem interval (PMI), is a fundamental yet complex challenge in forensic medicine. Forensic pathologists usually rely on classic signs, such as body temperature, rigor mortis, and livor mortis; however, these traditional methods are notoriously imprecise and easily influenced by environmental and individual factors [63]. Therefore, attempts have been made to use biochemical analyses of stable fluids, especially cerebrospinal fluid (CSF) or vitreous humor (VH), to develop a more reliable, multi-method approach to PMI determination [64].

In a relatively small sample of 35 individuals without nervous system pathology and with a known time since death, Cecchi et al. studied the concentrations of GFAP, S100B, and NSE in CSF and VH [65]. The mean PMI was 2.94 days with a range of 1–6 days. GFAP concentration increased gradually in CSF until 3 days of PMI, after which it decreased moderately. In most VH samples, GFAP could not be detected. NSE concentrations showed an irregular trend in both CSF and VH. S100B concentrations in CSF increased with the length of the PMI until day 5. The authors conclude that GFAP, NSE, and S100B concentrations in CSF may be promising biomarkers for PMI estimation, particularly in the early postmortem period within the first 2 days. The need for further studies, including more individuals, is clearly highlighted.

## 9. Brain morphology and behavior

Linking brain morphology to deviant or criminal behavior has a long, often controversial history, dating back to the crude phrenology of the 19th century. Charles Whitman, the "Texas Tower Sniper" who killed 16 people in 1966 before being shot by the police, left a suicide note requesting an autopsy to find a physical cause for his violent urges [66]. The post-mortem examination revealed a small mesencephalic-diencephalic tumor compressing his amygdala, a brain region critical for regulating aggression and fear [67]. While most experts involved in the case stressed that a relationship between Whitman's tumor and his actions cannot be established, the media nevertheless engaged in speculations about a direct impact of the tumor as the clear cause of the mysterious tragedy [68,69]. Since then, behavioral research in criminology, empowered by advanced neuroimaging technologies such as MRI and fMRI, has evolved into a modern neuroscience discipline.

In 2025, Kletenik et al. used structural brain imaging to examine brain morphology in 17 subjects with focal brain injuries associated with criminal behavior, identifying multiple white matter disconnections in the right uncinate fasciculus, forceps minor, corticostriatal tracts, and cingulum [70]. Their findings provide further lesion-based support for the existing literature on the important role of the right uncinate fasciculus, which connects the medial and orbital frontal cortices with the anterior temporal lobe and amygdala, regions known to be associated with emotion processing and to mediate reward-based processing and behavior. In addition, the lateralization of their findings provides further support for the existence of a right frontotemporal social-behavioral network. As brain imaging is increasingly used in courtrooms to support mitigating or exculpatory evidence, the authors caution that imaging findings alone are insufficient to establish causality.

Starčević and Ilić reviewed evidence on brain morphology in mass murderers, highlighting structural and functional abnormalities in regions critical for aggression, impulse control, and decision-making, particularly the prefrontal cortex, amygdala, orbitofrontal cortex, and hippocampus [71]. Neuroimaging studies often show reduced activity and smaller volumes in these regions. Neurotransmitter imbalances involving serotonin, dopamine, and norepinephrine may exacerbate these findings. These biological vulnerabilities, interacting with environmental factors such as trauma, may predispose individuals to extreme violence. While not deterministic, these findings may be important for prevention, intervention, and legal considerations, bridging neuroscience with criminological and judicial practice.

## 10. The neuropathology of alcohol use disorder

Alcohol use disorder (AUD) is defined as a problematic pattern of alcohol consumption that results in clinically significant impairment or distress, as evidenced by specific psychosocial, behavioral, or physiological criteria (the former terms "alcohol abuse" and "alcohol dependence" are no longer used) [72]. Chronic AUD is believed to alter brain chemistry and structure, driving addiction and increasing relapse risk. Because some individuals with AUD may develop disturbances that impair working memory and attention, similarities to neurodegenerative diseases have been noted, leading to the term alcohol-related brain damage (ARBD), which distinguishes these disturbances from changes due to nutritional deficiencies or hepatic failure. Early studies of ARBD found brain atrophy accompanied by reduced brain weight, most likely due to a potentially reversible loss of white matter [73]. Furthermore, neuronal loss in AUD has been described in the prefrontal cortex (PFC), a region associated with impulse control and decision making, thereby suggesting a possible morphological correlate for addictive behavior [74]. Fully characterizing the spectrum of ARBD is challenging because reliable clinical histories are often unavailable, and the clinical course is commonly confounded by co-occurring factors such as the use of other substances including illicit drugs, prescribed or over-the-counter medications, and tobacco, as well as systemic illnesses and nutritional deficiencies [73].

**Figure 8:**
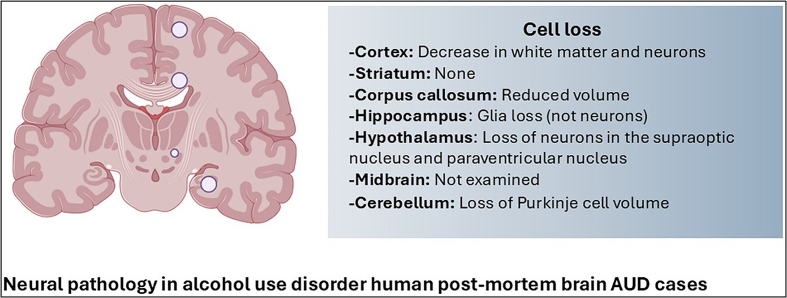
Overview of pathomorphological changes in the brains of individuals with AUD according to the review by Rasool et al. [75]. Reproduced under the Creative Commons Attribution 4.0 International License (http://creativecommons.org/licenses/by/4.0/).

For readers seeking deeper insight, the current state of knowledge on the neuropathology of AUD is presented in a comprehensive 2025 review by Rasool et al. [75]. The authors report that most brain regions studied in people with AUD show signs of damage and molecular changes that extend beyond local boundaries and affect broader brain networks. For example, in the PFC, degeneration primarily affects white matter, whereas in the hippocampus, a loss of glial cells may impair memory. In addition, a loss of neuropeptides in the hypothalamus could disrupt stress and reward systems, and degenerative changes in the cerebellum are believed to cause disturbances in coordination. Although there have been no reports of AUD-associated micro-morphological findings in the striatum, changes in certain neuron types in the ventral pallidum suggest molecular shifts in the basal ganglia that may drive alcohol-seeking behavior (**Figure 8**).

## Conclusion and outlook

In conclusion, this review of the 2025 literature shows that forensic neuropathology is evolving from a descriptive auxiliary discipline into a truly integrative field. It is no longer sufficient merely to document macroscopic findings; the modern forensic neuropathologist must synthesize classical observations with data from biomechanical models, advanced neuroradiology, genetic analysis, and bioinformatics. The studies highlighted here—from movement analysis of shaking events in AHT to the search for neuropathological correlates of IPV and the application of AI in postmortem imaging—illustrate this paradigm shift. Forensic neuropathology is moving toward a more mechanistic understanding of injury and disease, providing objective, reproducible evidence that withstands the strict scrutiny of both scientific and academic peer review and the courtroom. Ultimately, the future of forensic neuropathology lies in collaboration. Only closer communication with forensic pathologists, neuroradiologists, and bioengineers will build a strong evidence base. This approach will not only improve the accuracy of expert opinions in legal proceedings but also deepen our understanding of the fundamental mechanisms of brain injury and disease, transforming the "witness box" of the brain into a source of scientific insight.

## Disclosure

Generative artificial intelligence tools, specifically Grammarly and OpenAI's GPT-4, were used to prepare this manuscript. These tools helped refine sections of the text for the non-native English-speaking author. The final content has been reviewed and edited by the author to ensure accuracy, originality, and compliance with the journal's authorship and integrity guidelines. The author takes full responsibility for the manuscript's content.

## Conflict of interest statement

The author receives expert fees from investigative authorities and courts for preparing written or oral expert reports.
